# Supplementary material for: Pretreatment expression of miR-191a may predict response to the induction chemotherapy based on cytarabine in acute myeloid leukemia patients – a single-center pilotal study
Source: PLoS One. 2025 Jun 24;20(6):e0324320. doi: 10.1371/journal.pone.0324320 (PMC12186893; doi:10.1371/journal.pone.0324320)
Supplement: Attachment — Submitted filename: Review 1 microRNA PLOS ONE.docx [file pone.0324320.s002.docx]

Dear authors

The introduction is dense with information, which can make it difficult for readers to follow.

Some sentences are lengthy and contain multiple ideas, which may confuse readers.

Some information is repeated or could be consolidated.

The statistics provided regarding the incidence of AML could be presented more clearly. For example, stating the incidence rates in a comparative format would enhance clarity.

While the section states that patient characteristics are presented in Table 1, it does not provide any summary or highlights of these characteristics in the text.

The methodology for RNA isolation and analysis is described, but it lacks details about the quality control measures taken (e.g., RNA quality assessment) and how the specificity of the miRNA assays was ensured.

The statistical analysis section mentions using nonparametric tests due to the lack of normal distribution but does not provide any justification for the choice of tests used.

There is no justification provided for the sample size of 44 patients. A brief explanation of how this sample size was determined (e.g., power analysis) would strengthen the study’s methodology.

While the section states that patient characteristics are presented in Table 1, it does not provide any summary or highlights of these characteristics in the text.

There is little acknowledgment of potential methodological limitations within the study.

The discussion lacks a detailed description of the statistical methods used to analyze the correlations mentioned.
